# Gene expression analysis in SV-40 immortalized human corneal epithelial cells cultured with an air-liquid interface

**Published:** 2010-10-15

**Authors:** Dario Greco, Kati-Sisko Vellonen, Helen C. Turner, Marika Häkli, Timo Tervo, Petri Auvinen, J. Mario Wolosin, Arto Urtti

**Affiliations:** 1 Institute of Biotechnology, University of Helsinki, Helsinki, Finland; 2 Centre for Drug Research, University of Helsinki, Helsinki, Finland; 3 Division of Biopharmaceutics and Pharmacokinetics, University of Helsinki, Helsinki, Finland; 4 Department of Ophthalmology, Mount Sinai School of Medicine, New York, NY; 5 Department of Ophthalmology, University of Helsinki, Helsinki, Finland

## Abstract

**Purpose:**

To compare the global gene expression profile of stratified epithelia generated in vitro using simian virus 40 (SV40) immortalized human corneal epithelial cells with the previously reported gene expression of normal human corneal epithelia.

**Methods:**

Immortalized cells expanded in submerged culture were grown in an air-liquid interface of liquid permeable collagen-coated filters to foster stratification and differentiation. Stratified epithelia displaying resistances exceeding 300 Ω · cm^2^ were dissolved in an RNA purification lysis buffer. Purified RNA was used to globally determine gene expression levels using high-density single-channel oligonucleotide microarrays. Raw hybridization readings were converted into relative gene expression levels using Robust Multi-array Average (RMA) algorithm. Expression levels for selected genes were validated by real-time RT-qPCR. The biologic significance of the gene expression profiles was interpreted with the help of several microarray software analysis tools and ad hoc thematical analysis.

**Results:**

The stratified cell culture to native epithelial comparison identified over- and under-expression in 22% and 14% of the probed genes, respectively. The larger expression decreases occurred in genes intimately associated with both the stratified epithelial lineage at large such as keratin 14 and the corneal phenotype, such as keratin 12, connexin 43, aldehyde dehydrogenases (*ALDH*s), and paired box gene 6 (*PAX6*) and its whole downstream transcriptome. Overexpression related to genes associated with cell cycling stimulation.

**Conclusions:**

The results indicate that the stratified corneal epithelial cell model generated using SV40 immortalized cells may be useful only in certain research applications. Extrapolations of studies with these cells to actual tissue cells should be done with a great deal of caution.

## Introduction

The corneal epithelium is a stratified lining that serves as a critical protective barrier for the cornea. It prevents pathogen infiltration and limits fluid inflow into the transparent, dehydrated corneal stroma. The latter is primarily accomplished by high ionic resistance tight junctions coupled to an apical membrane with low solute permeability [1]. The junctions and the properties of the apical membrane develop as upwardly migrating cells reach the most apical position in a constant renewal process [2-5]. This barrier presents a challenge for the intraocular delivery of drugs and other medically useful compounds through the trans-corneal route. The stratified, compact nature of the lining also implies that applied compounds may be modified or metabolically eliminated and thereby not reach their intended intra-corneal or intra-ocular destinations.

In recent years, cell culture models based on both primary and immortalized cells have been developed as potentially reliable models of the native human corneal epithelium for either basic research or chemical testing [6]. This latter aspect reflect a need to find new means of ocular toxicity testing, which currently rely on undesirable ex vivo or in vivo animal experimentation (Draize test) [7]. A reliable in vitro human cell model of the corneal epithelium would reduce the need for such experiments and avoid the erroneous results that may originate from species differences.

Continuously growing cells are preferred as an indefinite source of human cells, because they are renewable and easily maintained. Simian virus 40 (SV40) immortalized human corneal epithelial (iHCE) cell lines were independently developed by Araki-Sasaki et al. [8] and Kahn et al. [9]. Immortalization is elicited by the expression in the transduced cells of the virus large T antigen, a master gene that causes global changes in gene expression [10]. These cells have been widely used in studies aimed at characterizing multiple activities or features of the corneal epithelium, including wound healing [11-13], gene transfer [14,15], drug transporters [16-18], cytotoxicity [19,20], and penetration properties of drugs [21].

In spite of the induced transformation, when grown on permeable filters at an air-liquid interface, the SV40 immortalized cells stratify, yielding multi-strata that resemble in many aspects both epithelia generated with untransformed corneal cells and native tissue [22]. Apical microvilli, tight junctions, and desmosomes can be easily identified. The model epithelia possess substantial electrical resistances, and their permeability to solutes approximates those of the native epithelium over a wide range of physicochemical properties [23]. Thus, tests with this model system may provide a viable alternative to investigating ocular absorption and toxicity in laboratory animals.

Yamasaki et al. [24] recently studied the genomic content of this cell line. They found that the genome of these cells is altered and contains several insertions and deletions compared to the normal genome. Since cell immortalization with large T antigen inhibits the function of tumor-suppressor proteins p53 and retinoblastoma 1, which contribute to the repair of DNA damage, genomic aberrations in the immortalized cells having high passage numbers (over 60) were not unexpected. Additionally, they investigated gene expression by the expressed sequence tags (EST) method and identified over 700 dominantly transcribed genes in the immortalized cells. A substantial fraction of genes encoding subunits of ribosomal proteins suggested enhanced protein synthesis in this cell line.

Since gene expression is strongly affected by cell culture conditions, we have now compared the gene expression profile of these cells when in the stratified, high transepithelial resistance condition, which is used to mimic the normal environment of the corneal epithelium, against the profile for the native, freshly isolated epithelium [22]. The results indicate that cells in the iHCE-based epithelium exhibits major differences in gene expression with respect to the reference tissue, particularly in regard to components of the tissue-specific phenotype.

## Methods

### Cell culture

The SV40 immortalized human corneal epithelial cell line (p4) was originally obtained from Dr. Hitoshi Watanabe (Osaka University, Osaka, Japan) [8]. During the cell expansion phase the iHCE cells were maintained in DMEM/Ham’s F12 (1:1; Gibco, Invitrogen, Paisley, UK), 15% FBS (Gibco, Invitrogen), 0.3 mg/ml L-glutamine (Gibco, Invitrogen), 5 µg/ml insulin (Gibco, Invitrogen), 0.1 µg/ml cholera toxin (Calbiochem, La Jolla, CA), 10 ng/ml EGF (Invitrogen, Carlsbad, CA), 0.5% dimethyl sulfoxide (DMSO; Sigma, St. Louis, MO), 0.1 mg/ml streptomycin, and 1000 IU/ml penicillin (Gibco, Invitrogen). Cells (passages of 22–23) were seeded on collagen-coated permeable supports (Transwell® Polyester Membrane Insert; Costar, Cambridge, MA) and cultured for 7 days as described earlier [22]. The medium was then supplemented with 40 µg/ml L(+)-ascorbic acid (Sigma, St. Louis, MO) and the supra-apical solution was removed. Trans-epithelial electrical resistance was tracked in situ with an EVOM resistance meter in Endohm chambers (World Precision Instruments, Sarasota, FL).

### Microarray processing

Cultures with resistances exceeding 300 Ω · cm^2^ were dissolved in TriReagent (MRC, Columbus, OH). Total RNA isolated from this solution was further purified using RNAeasy spin columns (Qiagen, Valencia, CA). RNA concentration and purity were determined from 260 nm and 280 nm absorbances. Integrity was determined using the Agilent 2100 BioChip (Agilent Technologies., Palo Alto, CA). The RNA was subjected to a single amplification run, labeled with biotin nucleotides, digested into proper size fragments, and hybridized to the HG-U133A gene microarray (Affymetrix, Santa Clara, CA) following a standard protocol established by Affymetrix. Hybridized chips were reacted with FITC-avidin and raw fluorescence intensities were read with a laser reader. HG-U133A contains >22,000 probes that provide for the representation of about one-half of the human genome. The raw signal intensity readings have been deposited in the Gene Expression Omnibus (GEO) under the accession number GSE22539.

The tissue (t)HCE data used in this study were generated previously for comparative study of gene expression profiles in freshly isolated human corneal and conjunctival epithelia [25]. The intact central cornea tHCE was obtained in that study by overnight incubation of quarters of donor cadaver corneas (procured from the National Disease Research Interchange (Philadelphia, PA) at 4 °C in 5 μg/ml Dispase type II dissolved in DMEM. The raw data in the form of an Affymetrix file can be found in the public domain GEO, series accession number GSE5543.

It is pertinent to point out that the experimental steps for the generation of the microarray results, starting with RNA repurification and ending in HG-U133A signal intensity readings, were performed at the MicroaArray Shared Facility of the Mount Sinai School of Medicine, New York, NY, under near identical conditions, including reagent used, technical personnel and automated microarray instrumentation.

### Data analysis

Microarray raw data files for three independent replicates of iHCE stratified cultures and previously published tHCE generated from Dispase-isolated epithelia that were processed in an identical manner to the current processing were imported into R v. 2.8.0 Bioconductor [26]. Custom CDF v. 10 was used to re-annotate the probes present on the HG-U133A chipset according to the Entrez gene database [27]. This reannotation considers only the microarray probe most proximal to the 3′end of the target sequence. Relative gene expression values were calculated by the Robust Multi-array Average (RMA) algorithm. In this method, normalization is performed across the whole data set; only the perfect match (PM) of the Affymetrix probes are used [28]. iHCE/tHCE ratios (Rs) are displayed throughout the tables as the logarithm on the base 2 of R.

Differential expression was tested by the *t*-test implemented in the limma package [29]. One set of over-, and under-expressed genes consisted of those genes complying with the p<0.01 criteria after application of the post hoc Benjamini-Hochberg correction, which allows a False Discovery Rate (FDR) <1%. A second, highly restricted set consisted of those genes complying with the p<0.01 filter after processing the data using the exacting Bonferroni post hoc correction.

The Database for Annotation, Visualization, and Integrated Discovery (DAVID) functional annotation tool [30] was used to identify over- and under-represented biologic themes. Gene networks were inferred using the Genomatix BiblioSphere v. 7.0 software. In the BiblioSphere process, connections in the network were drawn if two genes were either co-cited in the literature or contain consensus binding sites in their promoter regions for specific transcription factors and global differences in genes based on their promoters. In addition, differences in selected critical cell signal transduction pathways or gene families were manually examined using pathways depicted in Kegg or Biocarta.

### Real-Time RT–PCR

iHCE RNA was isolated from two separate cell culture batches, each with three replicates, distinct from those used for the microarray measurements. Three independent replicates of tHCE samples were obtained from photorefractive keratectomy (PRK) eye surgery performed at the Eye Clinic Silmäkeskus Laser Oy, Helsinki, Finland. Collection of this tissue was sanctioned by the local IRB and performed after obtaining informed, written consent from the donors. The use of human tissues was in accordance with the Declaration of Helsinki. Total RNA was isolated from these samples using RNAqueous^®^ -Micro or RNAqueous^®^-4PCR kits (Ambion, Austin, TX).

Quantitative real-time RT–PCR was used to validate the microarray results using a combination of over- and under-expressed genes. Genomic DNA contamination was eliminated by treating the samples with DNase I (Ambion). RNA (2 µg) was transcribed into cDNA using M-MuLV reverse transcriptase (Fermentas, Hanover, MD) and random primers (Fermentas). The PCR reaction was conducted in an ABI Prism 7000 instrument using TaqMan® Gene Expression Master Mix (Applied Biosystems, Foster City, CA) complemented with an amount of cDNA derived from 40 ng RNA and Taqman® Gene Expression Assays (Applied Biosystems; Table 1). For *ABCB1* and *ABCG2* genes, custom-made primers and probes described in Korjamo et al. [31] were used. Each sample was analyzed as triplicates and the relative levels of expression were calculated by the comparative cycle threshold method (ΔΔC_T_). Normalization was performed using the geometrical means of *TAF1C* (Hs00375863_m1) and *ABCB11* (Hs00184824_m1) C_T_s as normalizing values. Commonly used normalization genes, *ACTB* and *GAPDH*, have somewhat different expression levels in the iHCE and tHCE and thus these genes were considered as unsuitable for normalization. *TAF1C* and *ABCB11* genes had similar expression levels in iHCE and tHCE based on both microarray and real-time RT–PCR experiments. Therefore, these genes were chosen for normalization. Statistical significance was calculated using unpaired *t*-test.

**Table 1:** iHCE/tHCE expression ratio (R) of selected genes determined for microarrays (RMA method) or by real-time PCR

**Function**	**Symbol**	**Entrez gene ID**	**Microarray Log_2_ R (Adjusted p-value)**	**Real-time RT–PCR Log_2_ R (p-value)**	**TaqMan® gene expression assay**
Hair keratin	*KRT81*	3887	5.04 (1.20E-05)	7.87 (3.16E-07)	Hs00605559_m1
Hyaluronan-mediated motility receptor	*HMMR*	3161	4.51 (1.39E-06)	4.11 (5.67E-07)	Hs00234864_m1
Keratin of simple epithelia	*KRT7*	3855	3.84 (1.79E-04)	10.07 (2.42E-09)	Hs00818825_m1
Breast cancer resistance prot., stem cell related	*ABCG2*	9429	2.60 (1.38E-05)	6.43 (1.44E-08)	Custom-made*
MDR1; drug efflux pump	*ABCB1*	5243	1.54 (1.73E-03)	8.63 (1.04E-07)	Custom-made*
Protein phosphatase regulatory subunit	*SAPS3*	55291	0.00 (9.93E-01)	1.09 (1.01E-03)	Hs00217759_m1
Pore forming claudin	*CLDN15*	24146	−0.53 (4.30E-03)	0.81 (2.08E-02)	Hs00204982_m1
Receptor for hyaluronic acid	*CD44*	960	−0.99 (8.85E-03)	−0.43 (1.98E-01)	Hs00153304_m1
MRP5; drug efflux pump	*ABCC5*	10057	−1.37 (5.54E-04)	−1.72 (2.17E-05)	Hs00981071_m1
Component of tight junction strands	*CLDN1*	9076	−3.43 (8.43E-04)	−2.33 (1.31E-03)	Hs01076359_m1
Marker for corneal epithelial differentiation	*KRT3*	3850	−6.57 (1.23E-06)	−20.27 (1.17E-11)	Hs00365080_m1
Marker for corneal epithelial differentiation	*KRT12*	3859	−8.60 (5.29E-08)	−22.30 (1.34E-11)	Hs00165015_m1

The asterisk indicates described by Korjamo et al. [31].

## Results

### RNA and microarray quality tests and validation

High purity and integrity of the iHCE RNA were comparable to those obtained for the tHCE RNA [25]. The quality report produced by AffyQCReport R Package [32] and hierarchical clustering (Appendix 1) demonstrated that the microarray data were of good quality and that the data from iHCE and tHCE formed two separate groups. Overall, the microarray results correlated well with the results of RT–PCR analysis in their direction (Table 1). The PCR measurement consistently yielded, though, larger expression ratios than those reported by the microarray. This is a common observation [33] likely due to tendency of Affymetrix methodology to overestimate low intensity reading (i.e., a noise issue).

### Transcriptome differences

We took genes for which the p values in Benjamini-Hochberg corrected iHCE-tHCE comparisons were lower than 0.01 as differentially expressed. This limit led to the definition of 2,630 and 1,685 genes as over- or under-expressed in the iHCE, or 21.9% and 14% of the total of 12,029 re-annotated genes. Because RMA does not probe for the possibility that genes may be actually not expressed in the tissue, as done in MAS5 analysis, e.g [25], the number of relevant total and differential genes may actually be smaller, but the percentiles involved are likely to change only minimally.

Table 2 lists the number of differentially expressed genes as a function of iHCE-tHCE expression ratio intervals. Table 3 summarizes the results of DAVID analysis for the differentially expressed genes. The complete lists of DAVID functional annotation clustering of genes over- and under-represented in iHCE are provided in Appendix 2 and Appendix 3, respectively. The most over-represented gene ontology categories were primarily associated with the cell cycle, mitosis, and DNA metabolism. Under-representation occurred in gene categories related to development, differentiation, cell adhesion, and motility. Finally, Table 4 lists the most over- and under-expressed individual genes in descending order of expression ratio. The complete lists of differentially expressed genes by Benjamini-Hochberg and Bonferroni post hoc corrections are provided in Appendix 4 and Appendix 5, respectively.

**Table 2:** Number of differentially expressed genes represented by ratio

** **	**Number of genes (% of annotated genes)**	
**Log_2_ R**	**Over-expressed**	**Under-expressed**
≥ 4.0	75 (0.62)	87 (0.72)
3.0–3.99	107 (0.89)	89 (0.74)
2.0–2.99	339 (2.82)	161 (1.34)
1.5–1.99	426 (3.54)	202 (1.68)

**Table 3:** Selected over- and under-represented gene ontology (GO) terms in iHCE

**Term**	**Count**	**p value**
**Over-represented**		
GO:0005634~nucleus	937	2.08E-52
GO:0007049~cell cycle	278	2.64E-46
GO:0044237~cellular metabolic process	1369	3.46E-39
GO:0006259~DNA metabolic process	241	5.66E-32
GO:0006396~RNA processing	149	5.51E-27
GO:0005739~mitochondrion	246	4.94E-25
GO:0005694~chromosome	130	1.69E-24
GO:0000166~nucleotide binding	437	2.88E-19
**Under-represented**		
GO:0032502~developmental process	446	7.66E-26
GO:0007154~cell communication	500	1.01E-17
GO:0009653~anatomic struct. morph.	181	9.75E-16
GO:0007165~signal transduction	453	7.80E-15
GO:0030154~cell differentiation	254	3.78E-14
GO:0006928~cell motility	81	2.98E-11
GO:0031988~membrane-bound vesicle	68	3.06E-10
GO:0007155~cell adhesion	118	3.57E-09

**Table 4:** Transcripts with highest under- and overexpression in the iHCE.

**Full Name**	**Symbol**	**Entrez gene ID**	**Log_2_ R**
**Over-represented genes**			
ribonucleotide reductase M2	*RRM2*	6241	6.58
cyclin B1	*CCNB1*	891	5.58
maternal embryonic leucine zipper kinase	*MELK*	9833	5.54
aurora kinase A	*AURKA*	6790	5.53
discs, large (Drosophila) homolog-associated protein 5	*DLGAP5*	9787	5.38
GTP cyclohydrolase 1	*GCH1*	2643	5.34
Epithelial cell adhesion molecule	*EPCAM*	4072	5.34
Dickkopf homolog 1 (*X. laevis*)	*DKK1*	22943	5.25
cyclin-dependent kinase inhibitor 3	*CDKN3*	1033	5.12
topoisomerase (DNA) II alpha 170 kDa	*TOP2A*	7153	5.10
keratin 81	*KRT81*	3887	5.04
neuropilin (NRP) and tolloid (TLL)-like 2	*NETO2*	81831	5.02
NDC80 homolog, kinetochore complex component (*S. cerevisiae*)	*NDC80*	10403	5.02
centrosomal protein 55 kDa	*CEP55*	55165	4.97
ZW10 interactor	*ZWINT*	11130	4.94
interleukin 6 (interferon, beta 2)	*IL6*	3569	4.94
Forkhead box A1	*FOXA1*	3169	4.92
cell division cycle 20 homolog (*S. cerevisiae*)	*CDC20*	991	4.91
budding uninhibited by benzimidazoles 1 homolog beta (yeast)	*BUB1B*	701	4.89
ELOVL family member 5, elongation of long chain fatty acids	*ELOVL5*	60481	4.86
kinesin family member 23	*KIF23*	9493	4.82
meiosis-specific nuclear structural 1	*MNS1*	55329	4.81
geminin, DNA replication inhibitor	*GMNN*	51053	4.78
RAD51 associated protein 1	*RAD51AP1*	10635	4.75
thymidylate synthetase	*TYMS*	7298	4.74
kinesin family member 11	*KIF11*	3832	4.74
asp (abnormal spindle) homolog, microcephaly associated (Drosophila)	*ASPM*	259266	4.72
annexin A3	*ANXA3*	306	4.71
sarcoglycan, epsilon	*SGCE*	8910	4.69
epithelial cell transforming sequence 2 oncogene	*ECT2*	1894	4.64
kinesin family member 15	*KIF15*	56992	4.64
myxovirus (influenza virus) resistance 1, interferon-inducible protein p78	*MX1*	4599	4.62
v-myb myeloblastosis viral oncogene homolog (avian)-like 1	*MYBL1*	4603	4.59
kinesin family member 20A	*KIF20A*	10112	4.58
non-SMC condensin I complex, subunit G	*NCAPG*	64151	4.56
activated leukocyte cell adhesion molecule	*ALCAM*	214	4.55
hyaluronan-mediated motility receptor (RHAMM)	*HMMR*	3161	4.51
ISG15 ubiquitin-like modifier	*ISG15*	9636	4.50
nicotinamide N-methyltransferase	*NNMT*	4837	4.45
bone marrow stromal cell antigen 2	*BST2*	684	4.44
**Full Name**	**Symbol**	**Entrez gene ID**	**-Log_2_ R**
**Under-represented genes**			
keratin 14	*KRT14*	3861	8.93
aldehyde dehydrogenase 3 family, memberA1	*ALDH3A1*	218	8.72
keratin 12	*KRT12*	3859	8.60
gap junction protein, alpha 1, 43 kDa	*GJA1*	2697	8.46
chemokine (C-X-C motif) ligand 14	*CXCL14*	9547	8.37
chromosome 10 open reading frame 116	*C10orf116*	10974	8.28
keratin 5	*KRT5*	3852	8.23
aldehyde dehydrogenase 1 family, member A1	*ALDH1A1*	216	7.97
clusterin	*CLU*	1191	7.59
S100 calcium binding protein A4	*S100A4*	6275	7.47
keratin 24	*KRT24*	192666	7.46
desmoglein 1	*DSG1*	1828	7.29
cartilage acidic protein 1	*CRTAC1*	55118	6.88
mal, T-cell differentiation protein	*MAL*	4118	6.79
tripartite motif-containing 29	*TRIM29*	23650	6.71
paired box 6	*PAX6*	5080	6.60
keratin 3	*KRT3*	3850	6.57
chloride channel accessory 2	*CLCA2*	9635	6.23
HOP homeobox	*HOPX*	84525	6.17
desmocollin 3	*DSC3*	1825	5.99
crystallin, alpha B	*CRYAB*	1410	5.92
chloride channel accessory 4	*CLCA4*	22802	5.61
insulin-like growth factor binding protein 2, 36 kDa	*IGFBP2*	3485	5.61
secretoglobin, family 2A, member 1	*SCGB2A1*	4246	5.58
collagen, type XVII, alpha 1	*COL17A1*	1308	5.47
hepatic leukemia factor	*HLF*	3131	5.38
tripartite motif-containing 36	*TRIM36*	55521	5.36
keratin 15	*KRT15*	3866	5.32
keratin 4	*KRT4*	3851	5.32
v-kit Hardy-Zuckerman 4 feline sarcoma viral oncogene homolog	*KIT*	3815	5.09
cadherin 13, H-cadherin (heart)	*CDH13*	1012	5.08
calmodulin-like 3	*CALML3*	810	5.04
mal, T-cell differentiation protein-like	*MALL*	7851	5.04
uroplakin 1B	*UPK1B*	7348	5.01
PERP, TP53 apoptosis effector	*PERP*	64065	4.97
serpin peptidase inhibitor, clade F, member 1	*SERPINF1*	5176	4.96
lysophosphatidic acid receptor 6	*LPAR6*	10161	4.96
visinin-like 1	*VSNL1*	7447	4.87
LY6/PLAUR domain containing 3	*LYPD3*	27076	4,83
zinc finger, BED-type containing 2	*ZBED2*	79413	4.80

The stratified iHCE cell model was initially developed for drug permeability studies. Expression of drug transporter proteins and metabolizing enzymes determines the applicability of the cells in drug transport studies. These genes were examined more closely, and the over- and under-expressed genes are listed in Table 5. Both the under- and overexpressed gene lists include members from the same gene families, suggesting that expression must be investigated at the level of individual genes. The full data set is found in Appendix 4.

**Table 5:** Drug transporters and metabolizing enzymes.

**Full name**	**Symbol**	**Entrez Gene ID**	**Log_2_ R**
**Over-represented genes in iHCE**			
Solute carrier F. 2 (facilitated glucose transporter), M. 10	*SLC2A10*	81031	3.81
Solute carrier F. 7 (cationic amino acid transporter, y+ system), M. 5	*SLC7A5*	8140	3.61
Solute carrier F. 22 (organic cation/ergothioneine transporter), M. 4	*SLC22A4*	6583	3.00
ATP-binding cassette, sub-F. G (WHITE), M. 2	*ABCG2*	9429	2.60
Transporter 1, ATP-binding cassette, sub-F. B (MDR/TAP)	*TAP1*	6890	2.08
Cytochrome P450, F. 1, sub-F B, polypeptide 1	*CYP1B1*	1545	1.88
ATP-binding cassette, sub-F. B (MDR/TAP), M. 7	*ABCB7*	22	1.83
ATP-binding cassette, sub-F. C (CFTR/MRP), M. 4	*ABCC4*	10257	1.67
ATP-binding cassette, sub-F. C (CFTR/MRP), M. 3	*ABCC3*	8714	1.56
ATP-binding cassette, sub-F. B (MDR/TAP), M. 1	*ABCB1*	5243	1.54
Solute carrier F. 1 (glial high affinity glutamate transporter), M. 3	*SLC1A3*	6507	1.52
Solute carrier F. 16, M. One (monocarboxylic acid transporter 1)	*SLC16A1*	6566	1.40
Solute carrier F. 2 (facilitated glucose transporter), M. 3	*SLC2A3*	6515	1.32
Solute carrier F. 15, M. 3	*SLC15A3*	51296	1.19
Solute carrier F. 2 (facilitated glucose transporter), M. 6	*SLC2A6*	11182	0.89
Solute carrier F. 2 (facilitated glucose transporter), M. 8	*SLC2A8*	29988	0.80
**Full name**	**Symbol**	**Entrez Gene ID**	**-Log_2_ R**
**Under-represented genes in iHCE**			
Solute carrier F. 7 (cationic amino acid transporter, y+ system), M. 8	*SLC7A8*	23428	3.24
Solute carrier F. 2 (facilitated glucose transporter), M. 1	*SLC2A1*	6513	2.16
Solute carrier F. 22, M. 14	*SLC22A14*	9389	1.68
Solute carrier F. 2 (facilitated glucose/fructose transporter), M. 5	*SLC2A5*	6518	1.67
ATP-binding cassette, sub-F. B (MDR/TAP), M. 6	*ABCB6*	10058	1.38
ATP-binding cassette, sub-F. C (CFTR/MRP), M. 5	*ABCC5*	10057	1.37
ATP-binding cassette, sub-F. G (WHITE), M. 1	*ABCG1*	9619	1.29
Cytochrome P450, family 2, sub-F. C, polypeptide 18	*CYP2C18*	1562	1.26
ATP-binding cassette, sub-F. C (CFTR/MRP), M. 8	*ABCC8*	6833	1.15
Cytochrome P450, family 2, sub-F. C, polypeptide 19	*CYP2C19*	1557	1.01
Solute carrier F. 2 (facilitated glucose transporter), M. 9	*SLC2A9*	56606	1.01
Solute carrier F. 22, M. 17	*SLC22A17*	51310	0.99
Solute carrier F. 6 (proline IMINO transporter), M. 20	*SLC6A20*	54716	0.83
Cystic fibrosis transmemb. conductance regulator (ABC sub-F. C, M. 7)	*CFTR*	1080	0.73
Cytochrome P450, family 2, sub-F. C, polypeptide 9	*CYP2C9*	1559	0.70
Solute carrier F. 5 (sodium/glucose cotransporter), M. 1	*SLC5A1*	6523	0.66
Cytochrome P450, F. 1, sub-F. A, polypeptide 2	*CYP1A2*	1544	0.66
Solute carrier F. 22 (organic anion/urate transporter), M. 11	*SLC22A11*	55867	0.61
Solute carrier F. 6 (neurotransmitter transporter, betaine/GABA), M. 12	*SLC6A12*	6539	0.57
Solute carrier F. 7 (cationic amino acid transporter, y+ system), M. 4	*SLC7A4*	6545	0.52
Solute carrier F. 16, M. Four (monocarboxylic acid transporter 5)	*SLC16A4*	9122	0.49

In the table, F indicates family and M indicates member.

### Cell fate genes

**Figure 1:**
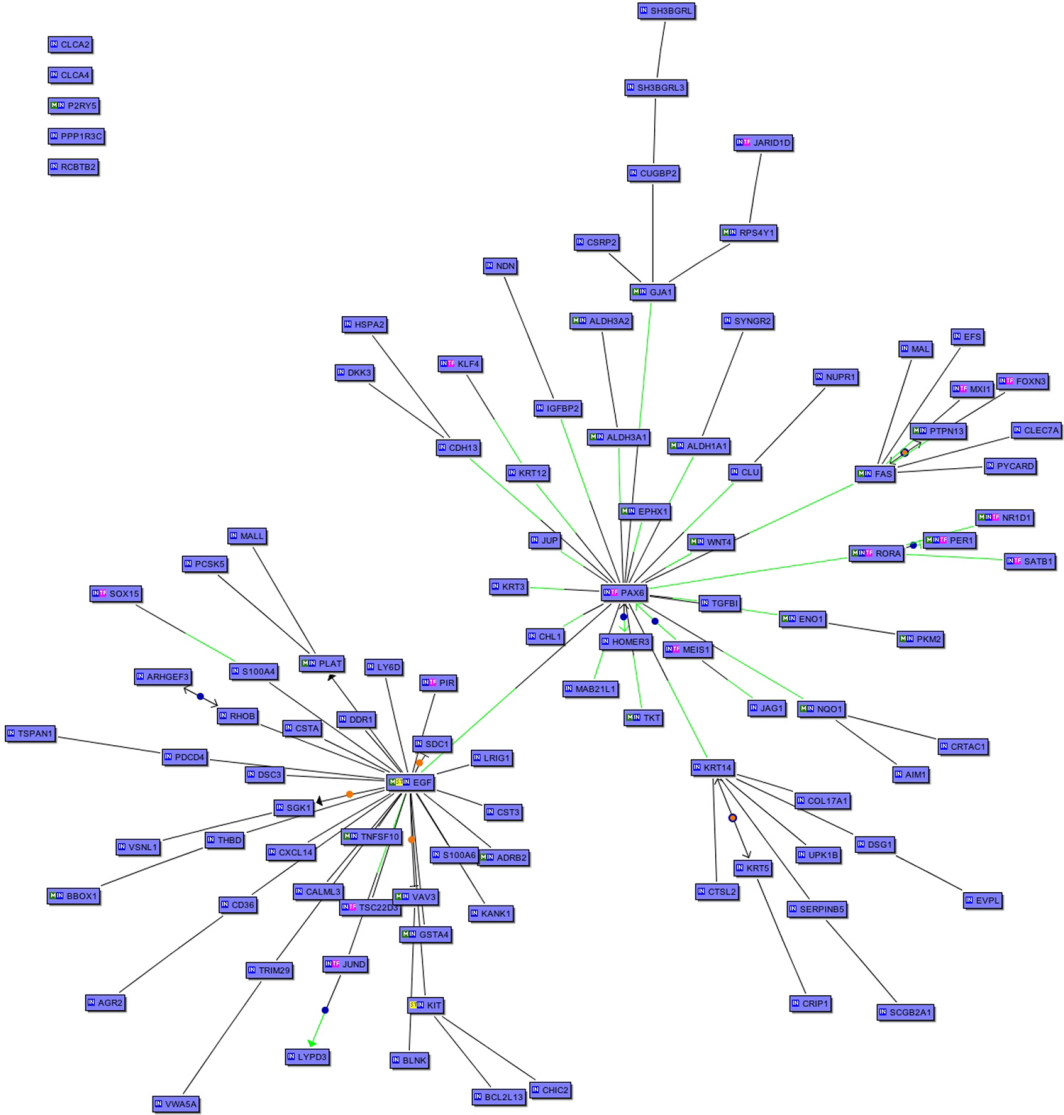
Network of the upregulated genes in the normal corneal epithelial tissue (p<0.01) after Bonferroni correction. Each box represents a gene; black edges represent co-citation and green edges indicate the binding of specific transcription factor on the gene promoter.

**Figure 2:**
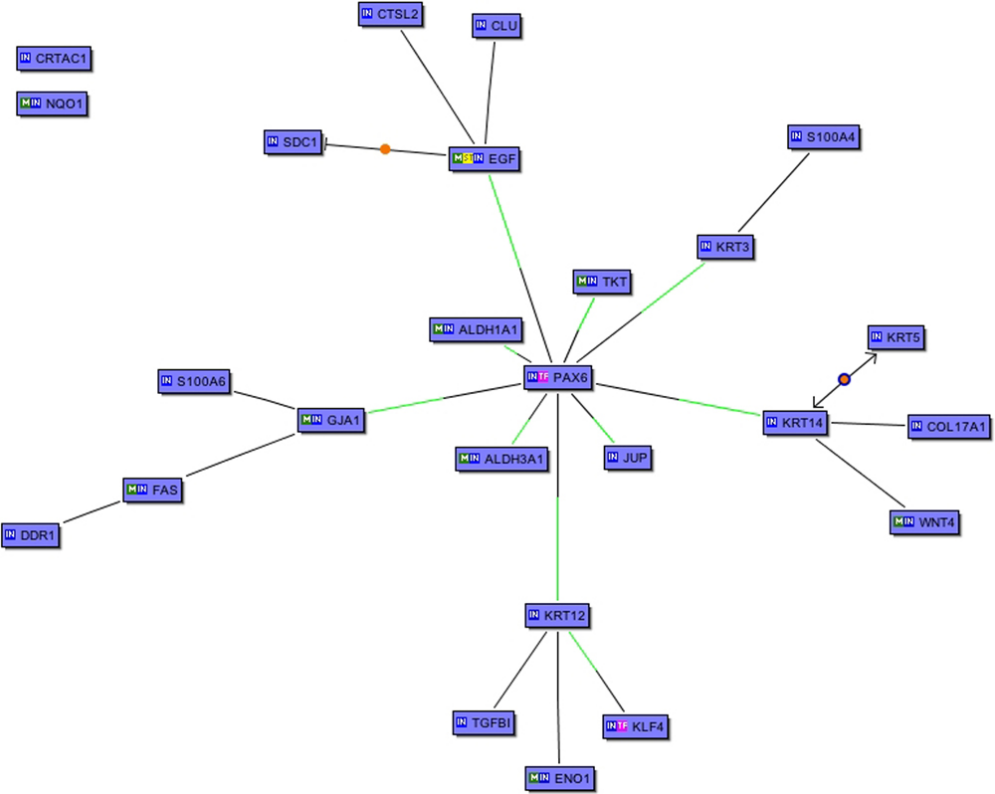
Network of the upregulated cornea-specific genes in the normal corneal epithelial tissue (p<0.01) after Bonferroni correction. Each box represents a gene; black edges represent co-citation and green edges indicate the presence of transcription factor binding of specific transcription factor on the promoter of the regulated gene.

Transcription factors and other genes acting as master genes for cell fate determine the overall pattern of gene expression of a cell. Thus, to identify the potential regulatory roots of the large expression differences between iHCE and tHCE, the subset of differentially expressed genes that complied with p<0.01 after applying the very exacting Bonferroni post hoc correction was used to develop gene-gene proximity maps with BiblioSphere. The Bonferroni compliant set consisted of 478 genes, 317 of which were under-expressed. Paired box gene 6 (*PAX6*) emerged from this analysis as the central gene, with possible binding sites on the promoters of several other genes in the tHCE (Figure 1). More detailed analysis of these promoters revealed a conserved module that is constituted by the consensus binding sites for PAX6 and BRN5 transcription factor families (Figure 2).

## Discussion

Reliable in vitro cell models are needed to mimic the human corneal epithelium. Such models should have a phenotype that maximally resembles the normal corneal epithelium. DNA microarrays enable a holistic analysis of gene expression, thus providing a powerful tool for comparing mortal, native tissue cells with transformed or immortalized cells which have been intentionally or spontaneously derived from the former and which may facilitate or accelerate research in the mother organ or tissue. The SV40 immortalized HCE cell line is widely used in ophthalmology.

In the present report, we have studied the gene expression in a stratified epithelium generated with the same cell line, but the cells were cultured on the semipermeable collagen coated membrane under airlift conditions to mimic the normal environment in the cornea. The original 22,000 plus Affymetrix reads of the HG-U133A chip were re-annotated using a sequence-based that has been shown to improve on the annotations provided by the microarray manufacture [27]. We have successfully used this approach in previous studies [34-36]. The robust computational methods applied revealed significant differences between the expression profiles of the transformed and parent human corneal epithelia. Upwards of 36% of the listed genes fitted the adopted definition for differential expression. Highly expressed corneal epithelial genes were related to the fundamental developmental processes. Cell-cell communication, cell adhesion, and differentiation were drastically repressed by the SV40 transformation process. Simultaneously, the expression of genes critically engaged in the control of cell division, in particular those associated with the G_2_/M progression and mitosis, underwent dramatic enhancements.

The changes in keratin expression profiles provide a robust, patent example of the large gene perturbation in terminal differentiation associated with the SV40 large T antigen effects [10]. Each stratified epithelium is defined by a distinct intermediate filament expression profile, and the corneal lining is characterized by the expression of its own keratin pair, keratin 3 (*KRT3*), and keratin 12 (*KRT12*) [37]. Respective to the in vivo expression of these two keratins, in the stratified SV40 cells expression was reduced by at least a hundredfold (Table 4). Previous studies have identified other genes undergoing similar changes in parallel to keratin, in particular connexin 43 and aldehyde dehydrogenase (*ALDH*) [38]. The strong de-expression of these two latter genes is a further confirmation of the immortalization process on tissue specific differentiation events (Table 4). The effects on phenotype, though, were not limited to those associated with the differentiated state. Multiple keratins associated with the undifferentiated state of stratified epithelial and even with their stem cells including *KRT4, KRT5*, *KRT14*, and *KRT15* [39] also underwent major reduction in expression following transformation while keratins of the simple epithelial cells (*KRT7* and *KRT18*) [39] became overexpressed. In summary, these results suggest that iHCE cells are ingrained with disturbances in their differentiation plan.

Mechanisms of gene regulation can be inferred from large gene expression studies by assuming that co-expressed or co-regulated genes might also be under the control of the same transcription factors [36]. The *PAX6* gene acts as the central master gene of eye morphogenesis. It is expressed in the corneal epithelium through development and adulthood. Its dosage is a critical determinant of migration, differentiation, and limbal stem cell function, where it determines critical behavior of the limbal-corneal stem cells [40-45]. Hence, the inadequate differentiation indicated by the keratin expression disturbance may originate in the absence of *PAX6* expression in iHCE cells. Interestingly, our analysis reveals that BRN5 might act as a co-regulator of *PAX6* in the corneal epithelium.

One of the main drivers for the development of iHCE lines was the need to establish in vitro models for corneal drug permeation studies [22]. The corneal epithelium is the main barrier that limits the absorption of topically applied ophthalmic drugs [46]. Stratified iHCE culture and ex vivo rabbit cornea showed similar paracellular space and passive permeability of 26 hydrophilic and lipophilic compounds [23]. The results of this study (Table 5) show dissimilar expression of membrane transporters and metabolic enzymes in the cell model and human corneal epithelium, respectively. This is in line with the recently published differences in the expression and functionality of monocarboxylate transporters [18] and ABC class efflux transporters [17] in the human corneal epithelium and cultured iHCE model. We should note, however, that the roles of membrane transporters and enzymes in ocular drug absorption are poorly understood.

Our recent literature analysis [47] revealed that 39 ocular drugs are known to be substrates to membrane transporters, but information about the expression and functionality of the transporters in the cornea is still sparse. Therefore, the impact of membrane transporters in the corneal drug absorption is unknown. Even though the DNA array analysis reveals differences in the transporter and enzyme expressions in the iHCE model and normal corneal epithelium (Table 5), there are no clear trends related to the families of transporters or enzymes. For example, both *ABC* and *SLC* transporters are found in the lists of overexpressed and under-expressed genes. Expression and functionality of transporter proteins should be further investigated and scaled to tissue properties before a stratified cell system based on the iHCE approach can be reliably applied to studies of active drug transport and metabolism.

The iHCE divergency in gene expression, though, may not occur or be so marked for features not associated with differentiation. Polarization and tightness of cell layers is a landmark of epithelial cell differentiation. The iHCE cell forms a tight permeation barrier with tight junctions and desmosomes shown at electron microscope level [22]. In this study, barrier properties of the cell model were confirmed by measuring transepithelial electrical resistance. Claudins 1, 4, and 11, which have been linked to the electric resistance and tightness of the cell barriers [48], were expressed at higher levels in the corneal epithelium than in the iHCE, but overall the expression differences for tight junction proteins were substantially less pronounced than those of the phenotype-associated markers, as were the genes coding for the desmosomal and cell-cell adhesion proteins desmoglein 1, desmoglein 3, desmocollin 3, and cadherin 13 [49] (Appendix 4). Finally, using the same microarray data analyzed in this report, Wang et al. [50] recently demonstrated a remarkable similarity of expression levels for most of the typical dual specificity phosphatases.

In conclusion, we demonstrated the differences in the global gene expression between the human corneal epithelium and stratified filter cultured cell culture system. Despite the correct morphology and barrier formation, there are still significant deviations of expression from the normal corneal epithelium. The SV40 transformed corneal epithelial cells could provide a useful model for certain areas of biologic study. However, the validity of the studies using these cells should be reconfirmed by parallel studies using native tissue or primary cells.

## Appendix 1. Quality report produced by AffyQCReport R Package and hierarchical clustering of iHCE and tHCE data.

To access the data, click or select the words “Appendix 1.” This will initiate the download of a compressed (pdf) archive that contains the file.

## Appendix 2. Functional annotation clustering of genes over-represented in iHCE.

To access the data, click or select the words “Appendix 2.” This will initiate the download of an Excel archive that contains the file.

## Appendix 3. Functional annotation clustering of genes under-represented in iHCE.

To access the data, click or select the words “Appendix 3.” This will initiate the download of an Excel archive that contains the file.

## Appendix 4. Differentially expressed genes by Benjamini-Hochberg correction.

To access the data, click or select the words “Appendix 4.” This will initiate the download of an Excel archive that contains the file.

## Appendix 5. Differentially expressed genes by Bonferroni post hoc correction.

To access the data, click or select the words “Appendix 5.” This will initiate the download of an Excel archive that contains the file.
